# Incarcerated Ink: A Case of Mycobacterium chelonae

**DOI:** 10.7759/cureus.58186

**Published:** 2024-04-13

**Authors:** Clayton F Staheli, Nicole N Dacy, Shannon C Brown, Palak Parekh

**Affiliations:** 1 Department of Dermatology, Texas A&M Health Science Center, Bryan, USA; 2 Department of Dermatology, Baylor Scott & White Medical Center - Temple, Temple, USA

**Keywords:** granulomatous reaction, incarceration, tattoo ink reaction, tattoo, nontuberculous mycobacterium, mycobacterium chelonae

## Abstract

A 30-year-old African American male presented with pain and swelling of the right foot one month after receiving a tattoo on this foot in prison. During his admission for presumed cellulitis, he developed a rash on his contralateral (left) leg, which had been tattooed 10 months prior. A biopsy of the contralateral (left) leg showed acute, chronic, and granulomatous inflammation with a differential diagnosis including infection. His overall condition and both legs worsened, prompting biopsy and tissue culture of the right ankle and foot. Pathology of the right foot showed a granulomatous reaction. Culture grew *Mycobacterium chelonae. *This case highlights the importance of considering infectious etiologies for rashes appearing within tattoos and represents the importance of a full investigation to obtain the correct diagnosis.

## Introduction

Tattooing is a common practice within the Western world, commonly reported as a way to commemorate a personal milestone or as a way to embellish one’s body [1]. Typically, infections resulting from tattooing have been caused by *Staphylococcus aureus* and beta-hemolytic streptococci [2]. However, reports have been increasing of infections due to *Mycobacterium chelona*e [2-4]. *M. chelonae* is a fast-growing, atypical, nontuberculous mycobacterium (NTM). It is highly drug-resistant as well as chlorine-resistant. Infections with *M. chelonae* can present in a myriad of ways, including soft tissue infections, abscesses, and disseminated disease. Cutaneous manifestations of an infection often follow traumatic injury, such as tattooing [5]. Due to the prevalence of *M. chelonae* in freshwater sources, dilutions of black ink to form a grey color have been associated with infections with this bacterium [2-4].

## Case presentation

A 30-year-old, incarcerated, African-American male presented to the emergency department with edema and pain in his right foot. The patient reported receiving a tattoo on this foot in prison one month prior to the presentation. His vitals were stable. Laboratory work-up was significant for a white blood cell count of 3.6 x 10^9^/L, erythrocyte sedimentation rate of 10 mm/hr (n = 0-15 mm/hr), and C-reactive protein level of 4.0 mg/dL (n = <0.9 mg/dL). Tests for HIV and hepatitis C were negative. MRI revealed soft tissue swelling without evidence of a drainable abscess. He was subsequently admitted for treatment of presumed cellulitis and started on IV vancomycin and ceftriaxone.

**Figure 1:**
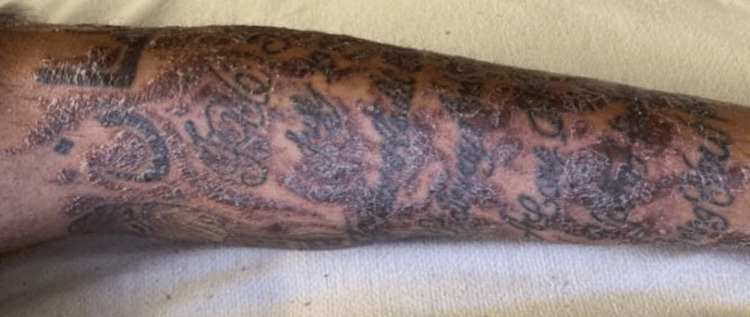
Left lower extremity rash

**Figure 2:**
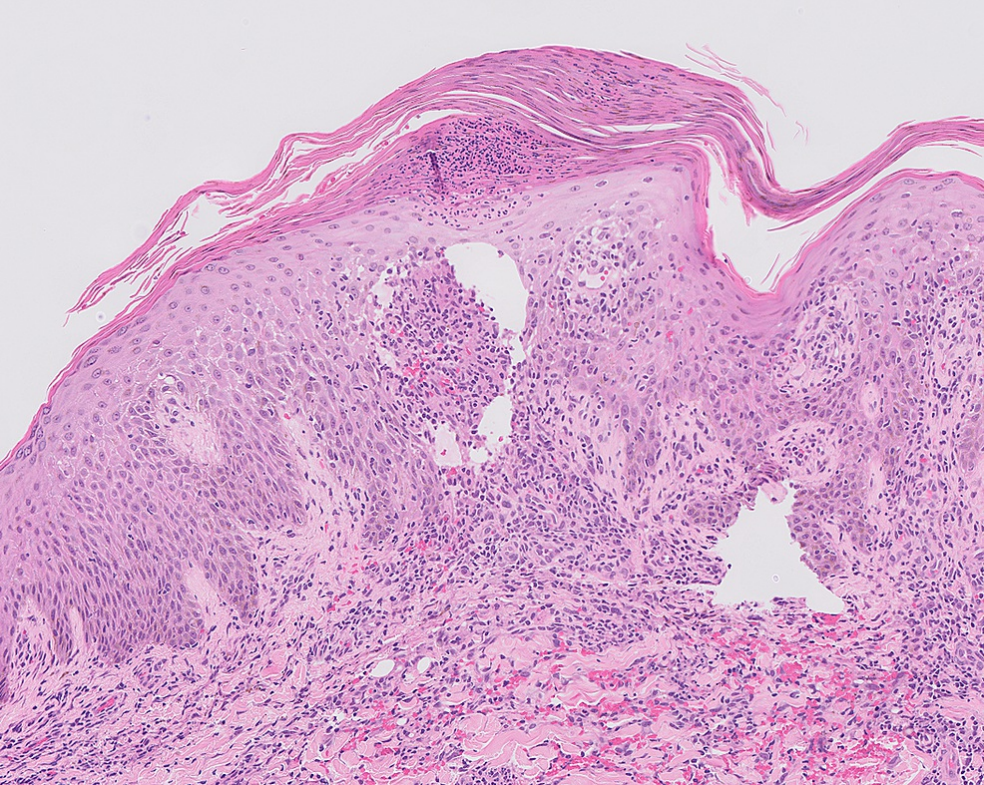
Spongiotic dermatitis with mixed inflammation of lymphocytes and neutrophils obtained from a punch biopsy of the left lower extremity

**Figure 3:**
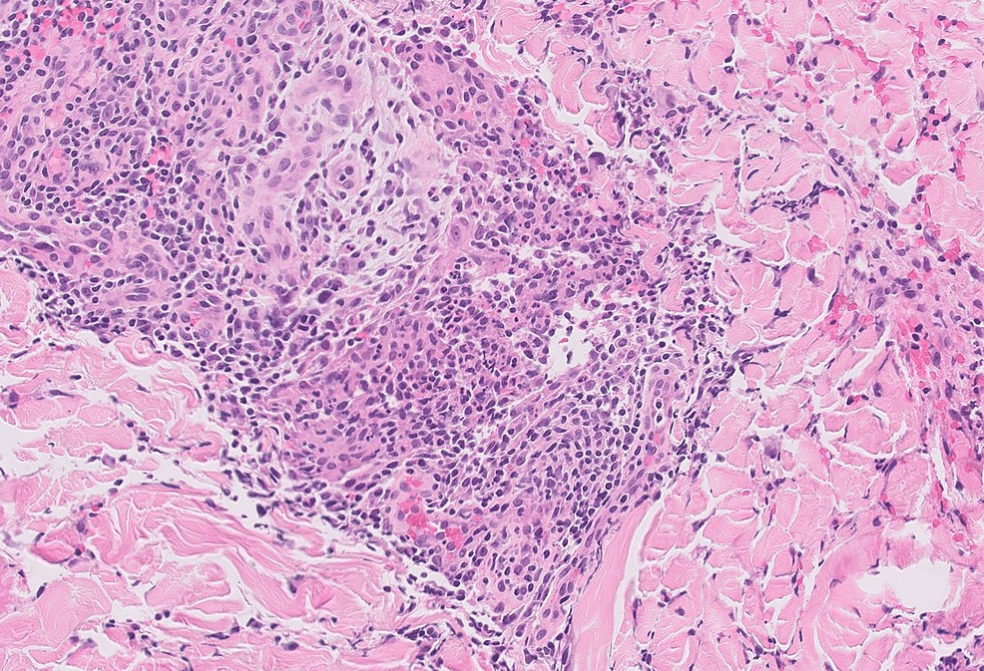
Granulomatous component of the left lower extremity punch biopsy

While admitted, the patient began to develop a reaction localized to a tattoo on his left lower leg (Figure 1). This tattoo was acquired 10 months prior to the presentation. Interestingly, he had numerous, previously acquired tattoos above the waist that were unaffected. A punch biopsy was performed of the left leg and revealed a spongiotic dermatitis with mixed inflammation containing mostly lymphocytes and small foci of neutrophils (Figure 2). A granulomatous component was also present. (Figure 3). These findings were concerning for an infectious etiology. The patient was discharged on IV vancomycin. The clinical differential for the left leg also included sarcoidosis and a tattoo reaction.

**Figure 4:**
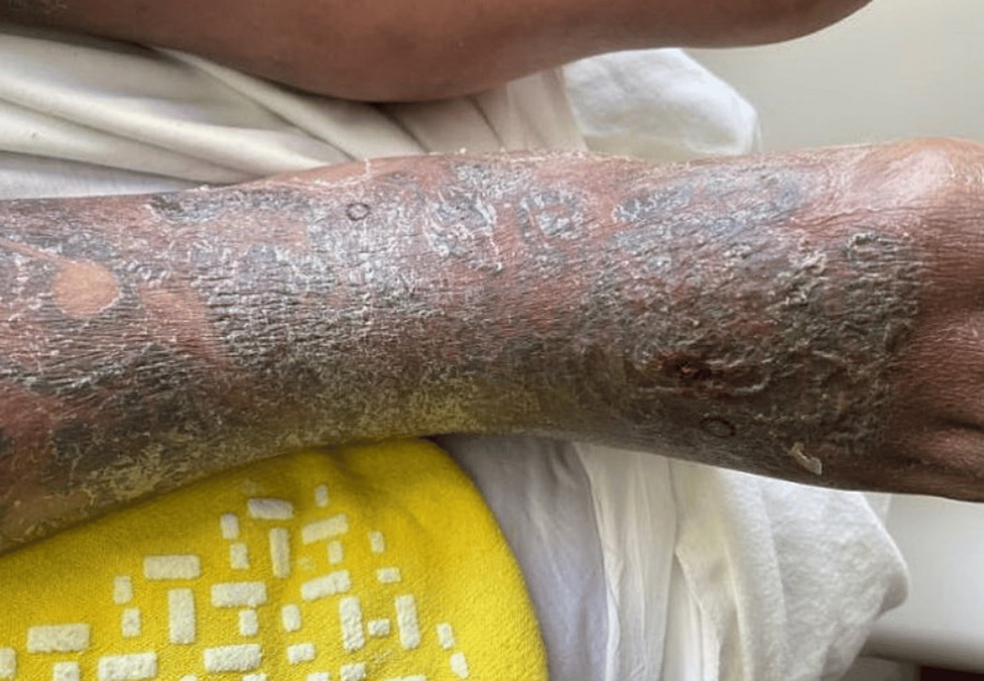
Edematous right foot at the time of re-admission

**Figure 5:**
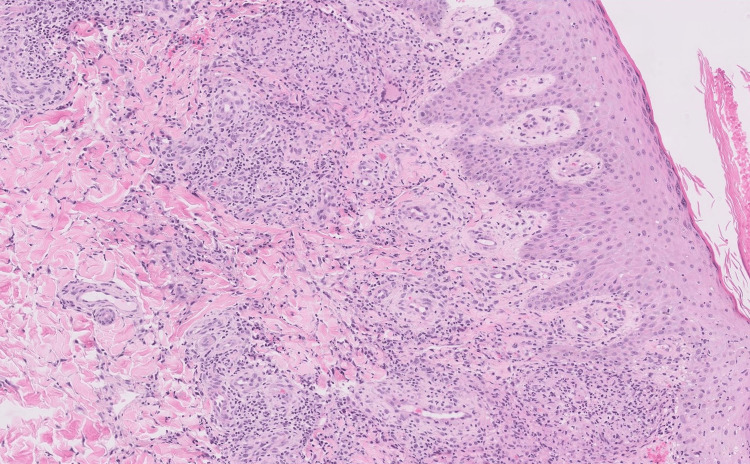
Second biopsy at the time of readmission displaying dermal granulomatous inflammation

One week later, he was re-admitted with worsening pain and edema of both lower extremities. Infectious disease was consulted, and antibiotics were broadened to linezolid and piperacillin-tazobactam. His right foot remained edematous, and purulence was noted (Figure 4). A biopsy for hematoxylin & eosin (H&E) and tissue culture were taken of the right medial ankle and right dorsal foot, respectively (Figure 5). The biopsy showed a mixed inflammatory cell infiltrate composed of granulomas, lymphocytes, and a few neutrophils. Pigment deposition in the papillary dermis was consistent with tattoo pigment. Special stains (periodic acid-Schiff, Grocott's methenamine silver, and acid-fast bacilli) were negative, suggesting a tattoo ink reaction. He showed improvement while inpatient and was again discharged on doxycycline for infectious coverage and a prednisone taper for possible tattoo ink reaction. Six days after discharge, tissue cultures from his medial right ankle grew *M. chelonae*. This strain of *M. chelonae* was resistant to doxycycline, cefoxitin, imipenem, moxifloxacin, and trimethoprim/sulfamethoxazole but susceptible to amikacin, linezolid, and tobramycin. The patient was lost to follow-up.

## Discussion

Complications arising secondary to tattooing must be thoroughly investigated as the differential diagnosis for a rash within a tattoo can be extensive. The differential includes, but is not limited to infection, suppurative granulomatous reactions, cell-mediated tattoo reactions, tuberculoid granulomatous reactions, sarcoidosis, and necrobiotic granulomatous reactions [6].

As a granulomatous disease, sarcoidosis presents microscopically as naked lymphocytic granulomas without neutrophils. These can appear in an isolated organ or systemically. These granulomas are often identified with inclusions such as asteroid bodies and Schaumann bodies [7]. Foreign body reactions in the skin, such as the introduction of tattoo ink, may also cause sarcoidal granulomas [8]. However, a distinguishing characteristic is pigmented granules present within the infiltrate. In the presence of a newly obtained tattoo and the absence of other systemic symptoms, this diagnosis can be considered more seriously.

With the granulomas characteristic of atypical mycobacterium, and more specifically *M. chelonae*, necrosis may occur, as opposed to sarcoidal granulomas. This, however, is not always the case and thus other histopathological features, such as neutrophilic microabscesses and curved acid-fast bacilli, may point toward the diagnosis of atypical mycobacterium infection [9]. Ultimately, the most definitive diagnostic tool is microbiotic culture, as even acid-fast bacillus (AFB) staining may be falsely negative, such as the presentation of our patient. The Löwenstein-Jensen (LJ) culture has previously been reported as having 79.5% sensitivity for the detection of all types of *Mycobacteria* species. This sensitivity, however, is increased to 91.7% when the LJ culture is combined with colorimetric detection methods such as the MB/BacT ALERT 3D System (BioMérieux, Marcy-l'Étoile, France) or to 95.5% when combined with fluorometric detection methods such as the BACTEC MGIT 960 System (Becton Dickinson, Franklin Lakes, NJ). Notable in this comparative evaluation study was the false negativity rate (71.4%) of atypical mycobacteria AFB smears [10]. A recent retrospective case series highlights this phenomenon of false negativity in AFB staining as only 11 of 73 (15.1%) of AFB stains were positive with a concomitant positive AFB culture [11]. It is imperative that clinicians retain a high index of clinical suspicion when encountering granulomatous reactions, and culturing such tissues may often be a key tool for the accurate diagnosis of a rash with a diverse differential.

Current guidelines advise treatment of *M. chelonae* with two of the following oral agents over the course of four months: (1) trimethoprim-sulfamethoxazole (1DS twice per day (BID)), (2) doxycycline (100 mg to 200 mg daily), (3) levofloxacin (500 mg daily), and (4) clarithromycin (500 mg BID) or azithromycin (250-500 mg daily); nevertheless, treatment needs to be guided by sensitivity results in cases of mycobacterial resistance [12,13].

## Conclusions

This case highlights the need for high levels of scrutiny when diagnosing rashes appearing in tattoos. The differential diagnosis is diverse with many diagnoses overlapping in their clinical appearance. Additionally, multiple external factors can contribute to rashes. This case exemplifies overlapping clinical and histological presentations of rashes arising in tattoos and highlights the importance of fully investigating the cause of the rash in the tattoo, including both biopsy and tissue culture, to obtain the correct diagnosis.
